# Rutaecarpine, an Alkaloid from *Evodia rutaecarpa*, Can Prevent Platelet Activation in Humans and Reduce Microvascular Thrombosis in Mice: Crucial Role of the PI3K/Akt/GSK3β  Signal Axis through a Cyclic Nucleotides/VASP—Independent Mechanism

**DOI:** 10.3390/ijms222011109

**Published:** 2021-10-15

**Authors:** Chun-Jen Huang, Wei-Chieh Huang, Wei-Ting Lin, Lan-Hsin Shu, Joen-Rong Sheu, Oanh-Thi Tran, Chih-Wei Hsia, Thanasekaran Jayakumar, Periyakali Saravana Bhavan, Cheng-Ying Hsieh, Chao-Chien Chang

**Affiliations:** 1 Department of Anesthesiology and Integrative Research Center for Critical Care, Wan Fang Hospital, Taipei Medical University, Taipei 110, Taiwan; cjhuang@tmu.edu.tw; 2 Graduate Institute of Clinical Medicine, College of Medicine, Taipei Medical University, Taipei 110, Taiwan; 3 Graduate Institute of Medical Sciences, College of Medicine, Taipei Medical University, Taipei 110, Taiwan; d119110003@tmu.edu.tw (W.-C.H.); m120108025@tmu.edu.tw (W.-T.L.); sheujr@tmu.edu.tw (J.-R.S.); d119106003@tmu.edu.tw (C.-W.H.); jayakumar@tmu.edu.tw (T.J.); 4 Department of Nutrition, Chung Shan Medical University, Taichung 402, Taiwan; s0743031@gm.csmu.edu.tw; 5 Department of Pharmacology, School of Medicine, College of Medicine, Taipei Medical University, Taipei 110, Taiwan; 6 International Ph.D. Program for Cell Therapy and Regeneration Medicine, College of Medicine, Taipei Medical University, Taipei 110, Taiwan; m142108001@tmu.edu.tw; 7 Department of Zoology, Bharathiar University, Coimbatore 641046, Tamil Nadu, India; bhavan@buc.edu.in; 8 Department of Cardiovascular Center, Cathay General Hospital, Taipei 106, Taiwan; 9 School of Medicine, College of Medicine, Fu Jen Catholic University, New Taipei City 242, Taiwan

**Keywords:** cyclic nucleotide, human platelets, hydroxyl radical, rutaecarpine, MAPK, microvascular thrombosis, PI3K/Akt/GSK3β, VASP

## Abstract

The role of activated platelets in acute and chronic cardiovascular diseases (CVDs) is well established. Therefore, antiplatelet drugs significantly reduce the risk of severe CVDs. *Evodia rutaecarpa* (Wu-Chu-Yu) is a well-known Chinese medicine, and rutaecarpine (Rut) is a main bioactive component with substantial beneficial properties including vasodilation. To address a research gap, we investigated the inhibitory mechanisms of Rut in washed human platelets and experimental mice. At low concentrations (1–5 μM), Rut strongly inhibited collagen-induced platelet aggregation, whereas it exerted only a slight or no effect on platelets stimulated with other agonists (e.g., thrombin). Rut markedly inhibited P-selectin expression; adenosine triphosphate release; [Ca^2+^]i mobilization; hydroxyl radical formation; and phospholipase C (PLC)γ2/protein kinase C (PKC), mitogen-activated protein kinase, and phosphoinositide 3-kinase (PI3K)/Akt/glycogen synthase kinase-3β (GSK3β) phosphorylation stimulated by collagen. SQ22536 (an adenylate cyclase inhibitor) or ODQ (a guanylate cyclase inhibitor) did not reverse Rut-mediated antiplatelet aggregation. Rut was not directly responding to vasodilator-stimulated phosphoprotein phosphorylation. Rut significantly increased the occlusion time of fluorescence irradiated thrombotic platelet plug formation. The findings demonstrated that Rut exerts a strong effect against platelet activation through the PLCγ2/PKC and PI3K/Akt/GSK3β pathways. Thus, Rut can be a potential therapeutic agent for thromboembolic disorders.

## 1. Introduction

Traditional Chinese herbs have long been used as vital remedies worldwide. Rutaecarpine (Rut) aceous plants, particularly *Evodia rutaecarpa* (the dried fruit of this plant is known as “Wu-Chu-Yu” in China), have long been in traditional Chinese medication for the treatment of gastrointestinal disorders, headache, amenorrhea, and postpartum hemorrhage [1]. Several alkaloids—including three major alkaloids, namely dehydroevodiamine, evodiamine, and Rut—have been identified in *E. rutaecarpa* and exhibit potent pharmacological activities. Rut is widely used to treat hypertension; its mechanism of action involves stimulating either the release of nitric oxide (NO) that leads to the activation of guanylyl cyclase [2] or the calcitonin gene-related peptide that in turn relaxes vascular smooth muscles and reduces peripheral resistance [3]. In addition, compared with other tissues, the brain tissue requires a higher amount of oxygen and is highly sensitive to low-oxygen conditions caused by ischemia. Yamahara et al. [4] reported that Rut exerted a potent antianoxic effect on a potassium cyanide-induced cerebral anoxia model. These findings indicate that Rut can have beneficial effects on cardiovascular activities.

Platelets, which are anucleated blood cells, play a central role in thrombotic and hemostatic processes. Intravascular thrombosis causes various cardiovascular and cerebrovascular diseases. Platelet adherence and aggregation are considered to be involved in the initiation of intraluminal thrombosis. When a blood vessel is damaged, platelets adhere to the disrupted surface, and the surface-adherent platelets release some biologically active constituents that trigger their aggregation [5]. Antiplatelet drugs can significantly reduce the risk of severe events such as ischemic stroke, myocardial infarction, and vascular death in patients with atherosclerotic vascular diseases.

Rut (40–200 µM) inhibited platelet aggregation in human platelet-rich plasma (PRP) stimulated by agonists (collagen, adenosine diphosphate (ADP), and arachidonic acid) [6]. In addition, Rut inhibited the formation of [^3^H]-inositol monophosphate, suggesting that the inhibition of platelet aggregation by Rut is mediated through the inhibition of phospholipase C (PLC) activation [7]. Furthermore, Rut (25 µg/g) could considerably decrease the death rate in ADP-induced acute pulmonary thromboembolic mice [8], indicating that Rut exerted an effective antiplatelet effect in vivo and thus might be a potential therapeutic agent for arterial thrombosis. However, few studies have examined the molecular mechanisms through which Rut exerts the antiplatelet effect. In the present study, we investigated the molecular mechanisms of Rut both in washed human platelets and experimental mice.

## 2. Results

### 2.1. Rut Inhibits Agonist-Stimulated Washed Human Platelets Aggregation

**Figure 1:**
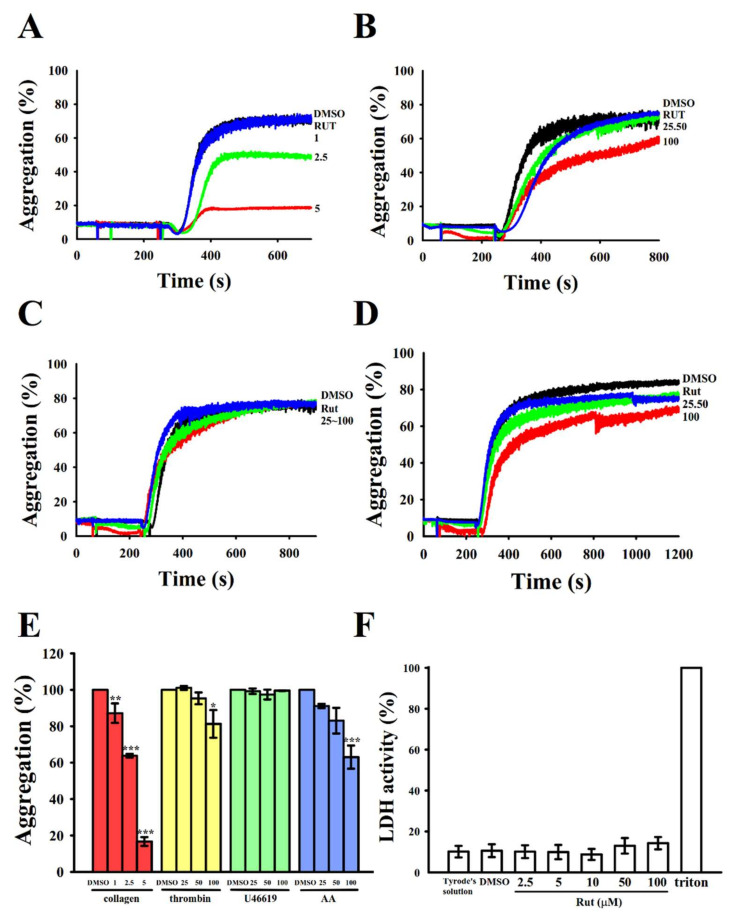
Inhibitory activities of rutaecarpine (Rut) on platelet aggregation and the cytotoxic test in human platelets stimulated with various agonists. Washed human platelets (3.6 × 10^8^ cells/mL) were preincubated with a solvent control (0.1% DMSO) or Rut (1–100 μM) and subsequently treated with collagen (**A**; 1 μg/mL), thrombin (**B**; 0.02 U/mL), U46619 (**C**; 1 μM), or arachidonic acid (AA) (**D**; 60 μM) to stimulate platelet aggregation. (**E**) Concentration–response histograms of Rut representing its inhibitory effects on platelet aggregation stimulated by various agonists (%). (**F**) To evaluate cytotoxicity, platelets were pretreated with solvent control (0.1% DMSO) or Rut (2.5,5, 10, 50, and 100 µM) for 20 min, and 10 µL of the supernatant was dropped on a Fuji Dri-Chem slide LDH-PIII. * *p* < 0.05, ** *p* < 0.01, and *** *p* < 0.001 compared with the 0.1% DMSO-treated group. Data (**E**,**F**) are presented as the mean ± standard error of the mean (*n* = 4).

As described previously [6], Rut (40–200 μM) significantly reduced platelet aggregation stimulated by various agonists in human PRP. However, except for that study, no study has reported any related evidence. The results of the current study revealed that Rut (1–5 μM) most strongly inhibited platelet aggregation stimulated by collagen (1 μg/mL) in washed human platelets (Figure 1A,E); however, it exerted only a slight or no effect on platelets stimulated with either thrombin (0.02 U/mL), arachidonic acid (AA; 60 µM), or U46619 (1 µM), a prostaglandin endoperoxide, or ADP (20 µM) (data not shown), even at concentrations up to 100 μM (Figure 1B–E). These results indicated that the efficacy of Rut differed among platelets stimulated with different agonists. The IC50 (2.5 µM) and maximal (5 µM) concentrations of Rut were subsequently employed to investigate the effects of Rut on collagen-stimulated human platelets. The findings of the lactate dehydrogenase (LDH) assay revealed that pretreatment with Rut (2.5–100 μM) for 20 min did not alter LDH release and exert any observable cytotoxic effects on platelets (Figure 1F), indicating that Rut did not cause significant cytotoxicity in platelets.

### 2.2. Regulatory Characteristics of Platelet Activation on Surface P-Selectin Expression, Adenosine Triphosphate–Release Reaction, and Relative [Ca^2+^]i Mobilization by Rut 

**Figure 2:**
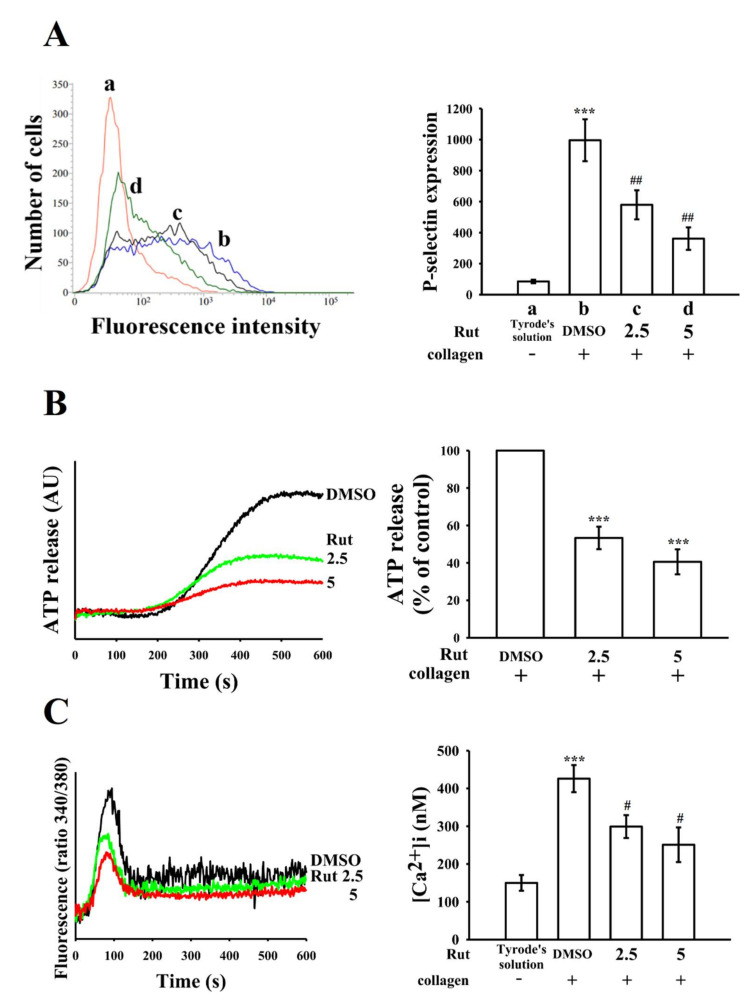
Inhibitory effects of Rut on surface P-selectin expression, adenosine triphosphate (ATP)-release reaction, and relative [Ca^2+^]i mobilization in human platelets. Washed platelets (3.6 × 10^8^ cells/mL) were preincubated with 0.1% DMSO or Rut (2.5 and 5 µM), followed by the addition of collagen (1 μg/mL) to trigger (**A**) surface P-selectin expression (a, resting control; b, collagen-activated; c, Rut 2.5 μM; d, Rut 5 μM), (**B**) ATP release (AU; arbitrary unit), and (**C**) relative [Ca^2+^]i mobilization, as described in the Materials and Methods section. The corresponding statistical data are presented in the right panel of each figure. Data are presented as the mean ± standard error of the mean (*n* = 4). (**A**,**C**) *** *p* < 0.001 compared with the resting control (Tyrode’s solution); ^#^ *p* < 0.05 and ^##^ *p* < 0.01 compared with the 0.1% DMSO-treated group. (**B**) *** *p* < 0.001 compared with the 0.1% DMSO-treated group.

The release of granular contents (in particular P-selectin from α-granules and adenosine diphosphate (ADP)/adenosine triphosphate (ATP) and Ca^2+^ from dense granules) are highly associated with platelet activation, thus resulting in an increase in platelet activation that can lead to a strong platelet aggregation. P-selectin is a crucial biomarker for platelet activation. Under normal conditions, P-selectin is expressed on the inner walls of α-granules; however, in an activated state, platelets expose the inner walls of the granules to the outer parts of the cells [9]. As indicated in Figure 2A, Rut markedly reduced surface fluorescein isothiocyanate (FITC)—P-selectin expression stimulated by collagen; the related statistical data is given in the right-hand panels of the figure (a, resting control, 84.8 ± 11.2; b, collagen-activated platelets, 995.8 ± 135.3; c, 2.5 μM Rut, 579.3 ± 93.8; d, 5 μM Rut, 361.2 ± 72.5; *n* = 4). In addition, Rut moderated ATP release upon collagen stimulation in a concentration-dependent manner (Figure 2B). In addition, increasing intracellular Ca^2+^ ([Ca^2+^]i) also plays an important role in platelet aggregation. Treatment with 2.5- and 5-μM Rut reduced the collagen-induced increase in [Ca^2+^]i by approximately 30% and 50%, respectively (resting, 150 ± 21 nM; collagen-activated platelets, 426 ± 36 nM; 2.5 μM Rut, 299 ± 30 nM; 5 μM Rut, 251 ± 46 nM; *n* = 4) (Figure 2C).

### 2.3. Effectiveness of Rut in PLCγ2/PKC Activation 

**Figure 3:**
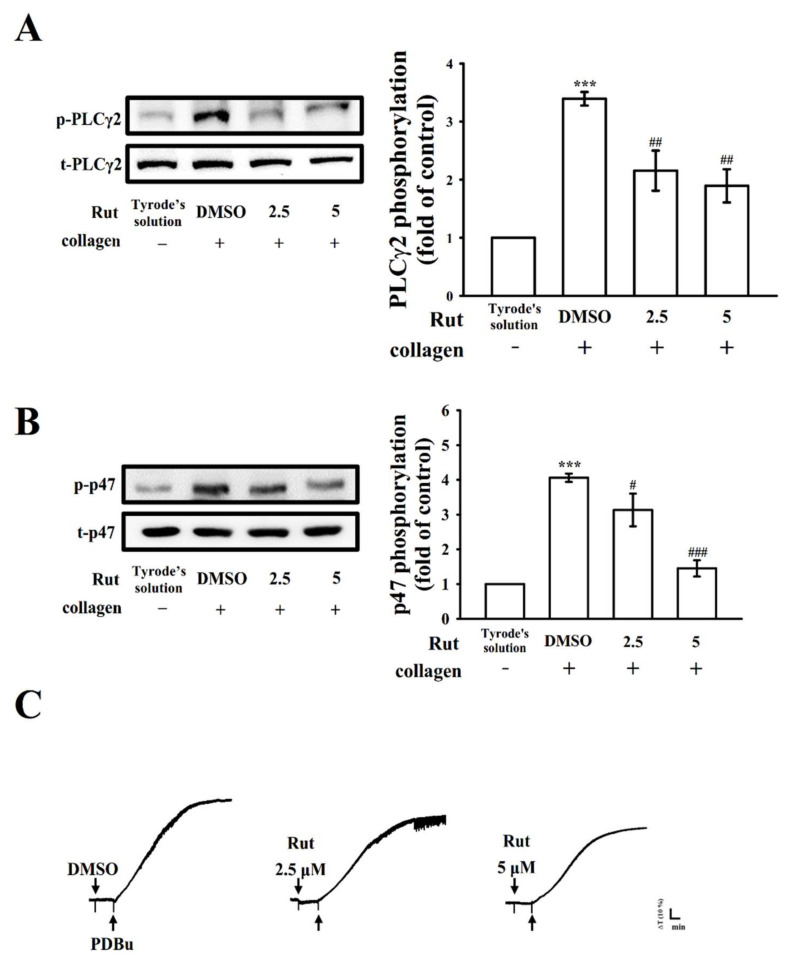
Regulatory effects of Rut on phospholipase Cγ2 (PLCγ2) and protein kinase C (PKC) activation in platelets. Washed platelets were preincubated with 0.1% DMSO or Rut (2.5 and 5 µM) and subsequently treated with collagen (1 µg/mL) or phorbol 12,13-dibutyrate (PDBu, 150 nM) to stimulate either (**A**) PLCγ2, (**B**) PKC activation (p-p47), or (**C**) platelet aggregation. Data are presented as the mean ± standard error of the mean (*n* = 4). *** *p* <0.001 compared with the resting platelets; ^#^ *p* < 0.05, ^##^ *p* < 0.01, and ^###^ *p* < 0.001 compared with the 0.1% DMSO-treated group. Profiles in (**C**) are representative of four independent experiments.

PLC, which belongs to a family of kinases, hydrolyzes phosphatidylinositol 4, 5-bisphosphate to produce two main secondary messengers, namely diacylglycerol (DAG) and inositol trisphosphate (IP_3_). DAG activates protein kinase C (PKC), triggering an approximately 47-kDa protein that is predominantly phosphorylated (pleckstrin or p47) and causes granule secretion, the main role of IP_3_ is to induce calcium mobilization [10]. Rut (2.5 and 5 µM) reduced PLCγ2 phosphorylation and PKC activation (p-p47) in collagen-activated platelets (Figure 3A,B). However, neither 2.5 nor 5 μM Rut significantly changed phorbol 12,13-dibutyrate (PDBu; a PKC activator)—induced platelet aggregation (Figure 3C), indicating that Rut did not directly exert its effect on PKC but on PLCγ2.

### 2.4. Regulatory Activity of Mitogen-Activated Protein Kinase and Phosphoinositide 3-Kinase-Akt-Glycogen Synthase Kinase-3β Activation by Rut

**Figure 4:**
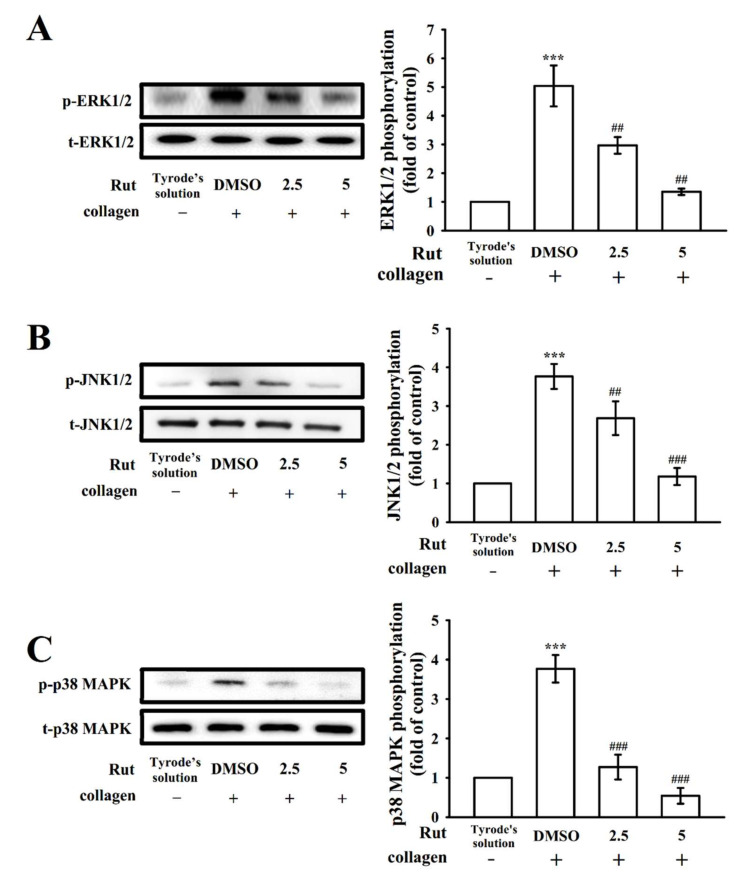
Regulatory characteristics of Rut on mitogen-activated protein kinase (MAPK) phosphorylation in platelets. Washed platelets were preincubated with 0.1% DMSO or Rut (2.5 and 5 µM) and then treated with collagen (1 μg/mL) for immunoblotting (**A**) ERK1/2, (**B**) JNK1/2, and (**C**) p38 MAPK phosphorylation. Data are expressed as the mean ± standard error of the mean (*n* = 4). *** *p* < 0.001 compared with the resting control; ^##^ *p* < 0.01 and ^###^ *p* < 0.001 compared with the 0.1% DMSO-treated group.

**Figure 5:**
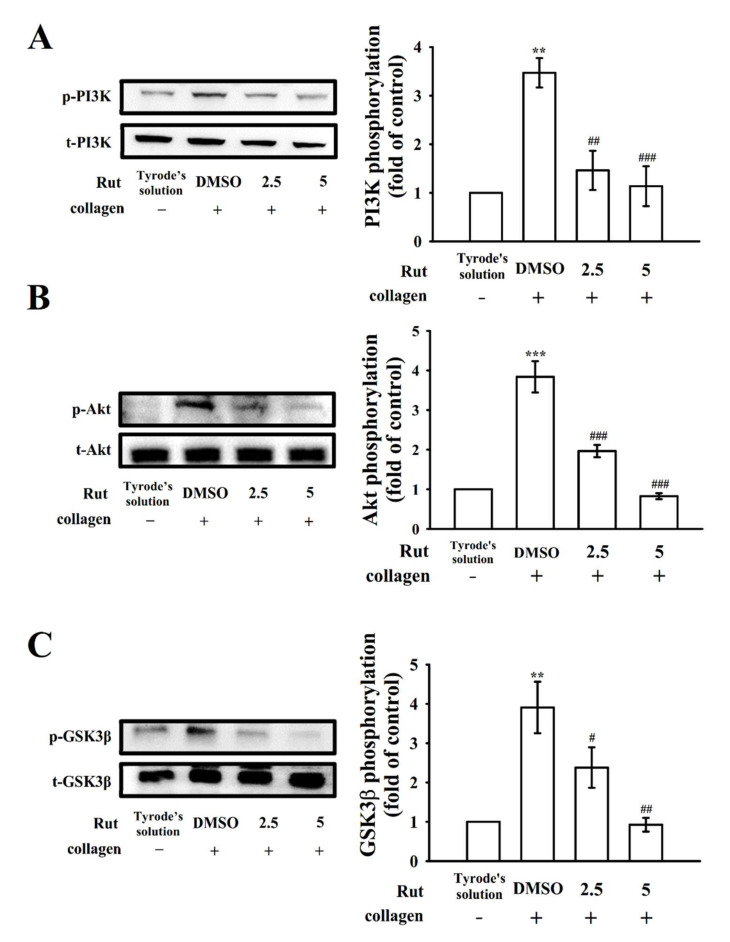
Effectiveness of Rut in PI3K/Akt/GSK3β phosphorylation in platelets. Washed platelets were preincubated with 0.1% DMSO or Rut (2.5 and 5 µM) and then treated with collagen (1 μg/mL) for immunoblotting (**A**) PI3K, (**B**) Akt, and (**C**) GSK3β phosphorylation. Data are expressed as the mean ± standard error of the mean (*n* = 4). ** *p* < 0.01 and *** *p* < 0.001 compared with the resting control; ^#^ *p* < 0.05, ^##^ *p* < 0.01, and ^###^ *p* < 0.001 compared with the 0.1% DMSO-treated group.

Mitogen-activated protein kinase (MAPK) signals regulate major cellular functions such as cell proliferation, apoptosis, inflammation, and platelet activation; MAPKs predominantly observed in platelets include extracellular signal-regulated kinase (ERK)1/2, Jun N-terminal kinase (JNK)1/2, and p38 MAPK [11]. Rut (2.5 and 5 µM) markedly reduced the phosphorylation of all three collagen-stimulated MAPKs, namely ERK1/2, JNK1/2, and p38 MAPK, demonstrating that MAPK signaling plays a crucial role in Rut-mediated antiplatelet activation (Figure 4). In addition, the phosphoinositide 3-kinase (PI3K)/Akt/glycogen synthase kinase-3β (GSK3β) pathway is involved in thrombus formation under high shear stress [12]. PI3K activation strongly contributes to platelet activation and is a major regulator of Akt activation [12]. Akt, referred to as protein kinase B (Ser/Thr kinase), can be activated by several platelet agonists that regulate platelet activation and hemostasis; GSK3β is a classical enzyme regulated downstream of the PI3K/Akt pathway in platelets [13]. In the current study, at concentrations of 2.5 and 5 μM, Rut markedly inhibited the phosphorylation of the PI3K/Akt/GSK3β pathway under collagen stimulation (Figure 5). These results indicate that the inhibition of MAPKs and the PI3K/Akt/GSK3β pathway may contribute critically to antiplatelet effects of Rut.

### 2.5. Role of Intracellular Cyclic Nucleotides in the Antiplatelet Effect of Rut

**Figure 6:**
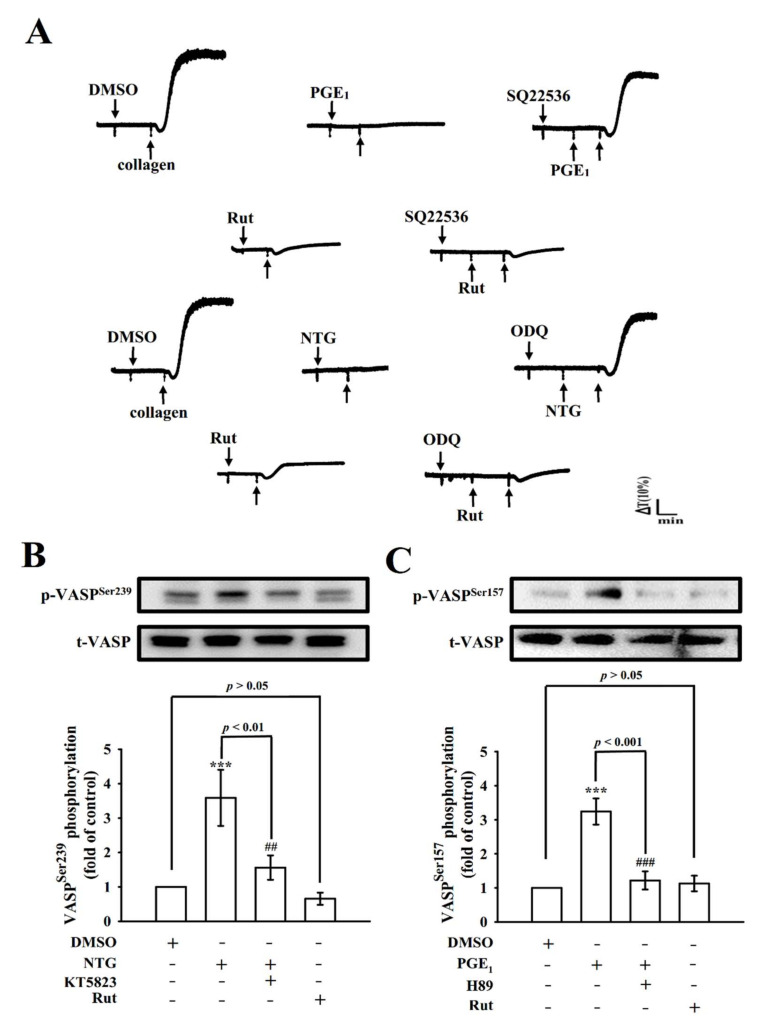
Effect of Rut on cyclic nucleotides and vasodilator-stimulated phosphoprotein (VASP) phosphorylation in human platelets. (**A**) Washed platelets (3.6 × 10^8^ cells/mL) were preincubated with (**A**) prostaglandin E_1_ (PGE_1_; 20 nM), nitroglycerin (NTG; 20 µM), or Rut (5 μM) in the presence of SQ22536 (50 μM) or ODQ (10 µM) for 3 min before the addition of collagen (1 μg/mL) to stimulate platelet aggregation. (**B**) Washed platelets were directly stimulated with 0.1% DMSO, NTG (20 µM), KT5823 (10 µM), or Rut (5 μM) for immunoblotting of VASP^Ser239^ phosphorylation. (**C**) Washed platelets were directly stimulated with 0.1% DMSO, PGE_1_ (20 nM), H89 (100 µM), or Rut (5 µM) for immunoblotting of VASP^Ser157^ phosphorylation. Profiles in (**A**) are representative of four similar experiments. Data are expressed as the mean ± standard error of the mean (*n* = 4). *** *p* < 0.001 compared with the 0.1% DMSO-treated group; ^##^ *p* < 0.01, and ^###^ *p* < 0.001 compared with the NTG- or PGE_1_-treated group.

Chiou et al. [14] reported that Rut caused vasodilation by releasing NO from endothelial cells; however, the antiplatelet activity of Rut was not significantly changed after pretreatment with NO synthase inhibitors such as N^G^-nitro-L-arginine methyl ester [6]. Cyclic nucleotides are small cyclic monophosphates, such as cyclic adenosine monophosphate (cyclic AMP) and cyclic guanosine monophosphate (cyclic GMP), these play as key secondary messengers in several signal transduction pathways responsible in the regulation of multiple targets including various protein kinases that participate in the phosphorylation of vasodilator-stimulated phosphoprotein (VASP). As shown in Figure 6A, SQ22536 (50 μM; an adenylate cyclase inhibitor) and ODQ (10 μM; a guanylate cyclase inhibitor) reversed the prostaglandin E_1_ (PGE_1_; 20 nM)- and nitroglycerin (NTG; 20 μM)-mediated inhibition of platelet aggregation stimulated by collagen, respectively; neither SQ22536 nor ODQ could reverse Rut (5 μM)-mediated antiplatelet activity. Moreover, NTG (20 μM) and PGE_1_ (20 nM) triggered the phosphorylation of VASP^Ser239^ (Figure 6B) and VASP^Ser157^ (Figure 6C), respectively. Treatment with KT5823 (a protein kinase G inhibitor; 10 μM) and H89 (a protein kinase A inhibitor; 100 μM) markedly reduced VASP^Ser239^ and VASP^Ser157^ phosphorylation (Figure 6B,C), respectively. However, Rut (5 μM) exerted no significant effect on both VASP phosphorylations, indicating that Rut has an antiplatelet mechanism without involvement of cyclic nucleotide in washed human platelets.

### 2.6. Effectiveness of Rut in Hydroxyl Radical Formation in Human Platelets and Microvascular Thrombosis as well as Tail Bleeding Time in Mice 

**Figure 7:**
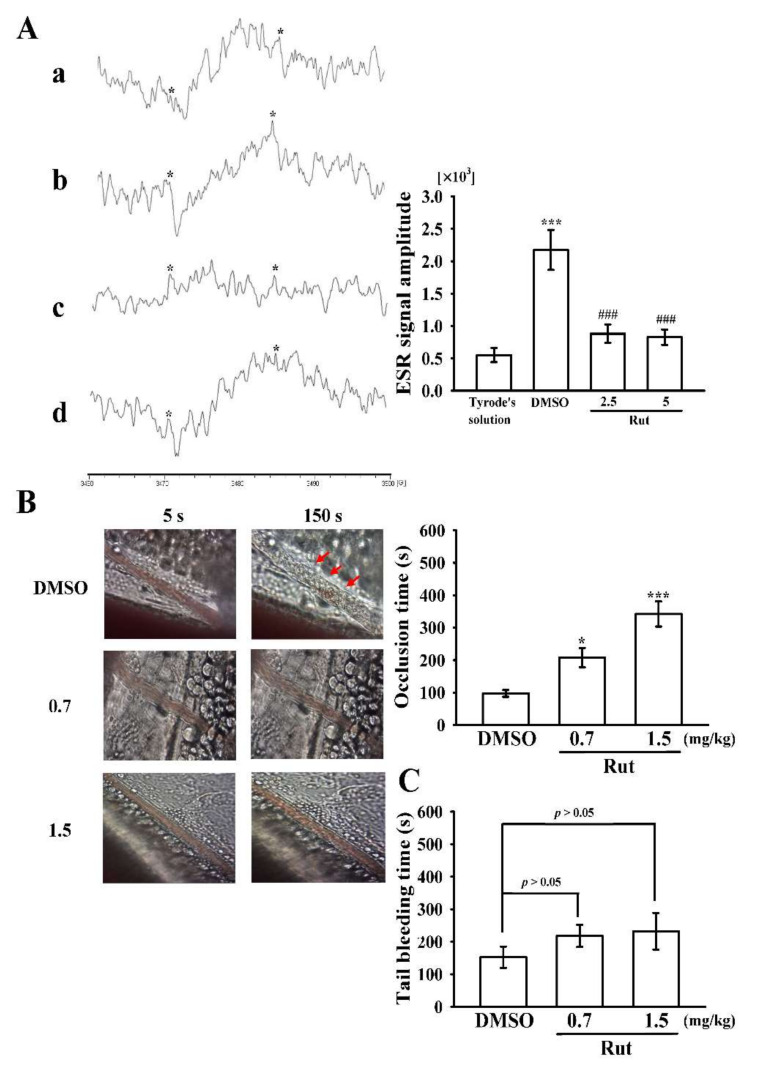
Inhibitory effect of Rut on hydroxyl radical formation in human platelets and microvascular thrombus formation as well as tail bleeding time in mice. (**A**) Washed platelets (3.6 × 10^8^ cells/mL) were preincubated with (a) Tyrode’s solution only (resting group) or preincubated with (b) 0.1% DMSO or Rut (c, 2.5; d, 5 µM); subsequently, collagen (1 μg/mL) was added to trigger hydroxyl radical (*) formation. Profiles are representative of four similar experiments. (**B**) Mice were administered an intraperitoneal bolus of the solvent control (0.1% DMSO) or Rut (0.7 and 1.5 mg/kg), and the mesenteric venules were irradiated to induce microthrombus formation (occlusion time). Microscopic images (400× magnification) of the 0.1% DMSO-treated group and Rut (0.7 and 1.5 mg/kg)-treated group were recorded at 5 and 150 s after irradiation, respectively. The photographs shown are representative of eight similar experiments, and red arrows indicate platelet plug formation. (**C**) The bleeding time was measured through mouse tail transection after 30 min of intraperitoneal administration of 0.1% DMSO or Rut (0.7 and 1.5 mg/kg). Data are presented as the mean ± standard error of the mean (**A**, *n* = 4; **B**,**C**, *n* = 8). **A**, ***p < 0.001, compared with the Tyrode’s solution only (resting group); ^###^ *p* < 0.001, compared with the 0.1% DMSO-treated group. **B**, * *p* < 0.05 and *** *p* < 0.001 compared with the 0.1% DMSO-treated group.

Reactive oxygen species (ROS), such as hydrogen peroxide and hydroxyl radicals, generated from the activated platelet might enhance platelet activity during thrombus formation. However, the controlling pathways of ROS, particularly for hydroxyl radicals, during platelet activation remain ambiguous. A typical electron spin resonance (ESR) signal of a hydroxyl radical was stimulated by collagen (1 μg/mL) and compared with that of resting platelets (Figure 7A(a), Tyrode’s solution, 551 ± 109; b, collagen activated, 2174 ± 308; *n* = 4). Rut (2.5 and 5 μM) markedly reduced hydroxyl radical formation stimulated by collagen (Figure 7A(c), Rut 2.5 μM, 881 ± 143; d, Rut 5 μM, 827 ± 118; *n* = 4). 

The therapeutic effect of the antithrombotic activity of Rut was examined in terms of prolonging the occlusion time (OT) in mice (Figure 7B). The OT in the mesenteric microvessels of mice pretreated with fluorescein sodium (15 µg/kg) was approximately 100 s. The OT was significantly prolonged after treatment with 0.7 or 1.5 mg/kg Rut compared with after treatment with 0.1% dimethyl sulfoxide (DMSO; solvent control, 97.4 ± 10.5 s vs. 0.7 mg/kg Rut, 207.9 ± 30.2 s; 1.5 mg/kg Rut, 342.3 ± 38.8 s; *n* = 8, Figure 7B). After irradiation, a thrombotic platelet plug was observed in the mesenteric microvessels at 150 s but not at 5 s in the group treated with 0.1% DMSO (Figure 7B; red arrows). However, in the group treated with Rut (0.7 and 1.5 mg/kg), platelet plug formation was not observed at either 5 or 150 s after irradiation (Figure 7B).

Moreover, we investigated the bleeding time through mouse tail transection 30 min after Rut and DMSO were intraperitoneally administered; the bleeding times were 156 ± 33 s (0.1% DMSO-treated group; *n* = 8), 218 ± 34 s (0.7 mg/kg Rut-treated group; *n* = 8), and 232 ± 56 s (1.5 mg/kg Rut-treated group; *n* = 8) (Figure 7C). To detect any rebleeding, mice were individually monitored for 15 min, even after the bleeding had stopped. The results suggested that Rut reduced platelet plug formation without significantly prolonging bleeding time.

## 3. Discussion

Short- or long-term administration of bioactive alkaloids exerted antiangiogenic, antiatherogenic, and antithrombotic effects on patients with cardiovascular diseases (CVDs) [15]. Rut could enhance atrial contraction, increase the contraction frequency, and protect the myocardium against ischemia-reperfusion injury; it also exerted a hypotensive effect by activating vanilloid receptor subtype 1 [16]. In terms of pharmacokinetics, Rut was rapidly absorbed after oral administration in rats, and its Tmax was approximately 0.5 h [17]. Because a low concentration of Rut (2.5 and 5 µM) was used in platelet suspensions while compared with PRP as described previously [6], indicating Rut may have a high protein (i.e., albumin) binding capacity which will prolong the retention time in the circulation. It likely would have reached the required plasma concentration at a reasonable time after its administration. Although normal Rut obtained from natural sources would be insufficient to achieve the required concentration of inhibiting platelet activation in vivo, its long-term consumption is ideal for preventing atherothrombotic events, especially in Chinese medicine with long-term therapeutic applications. Thus, Rut can be used as an effective antithrombotic drug in humans.

Rut effectively inhibited collagen-induced platelet aggregation but exerted no or only a slight effect on platelets stimulated by other agonists. This finding implies that the anti-platelet effect of Rut may occur via one of the important signals of PLC-dependent mechanism. The stimulation of platelets with agonists, such as collagen, could modify phospholipase activation. The activation of PLC leads to the formation of IP_3_ and DAG, which in turn activate PKC and subsequently induce the phosphorylation of p47 protein [18]. The PLCγ family consists of isozymes 1 and 2, and PLCγ2 participates in collagen-dependent signaling in platelets [19]. The PKC family comprises several isoforms that play a central role in serine/threonine phosphorylation events in most cell types; the activity of these cell types strongly affects various signal transduction events [10]. In the current study, Rut reduced collagen-activated PLCγ2/PKC activation; but it did not directly effect PKC activation as there is no reduction on PDBu-induced platelet aggregation. This finding suggests that PLCγ2 downstream signaling plays role on Rut-mediated inhibition of platelet activation. In addition, a series of tyrosine kinase cascades involve platelet activation and induce an increase in intracellular calcium and granule secretion (i.e., P-selectin and ATP). α-Granules are mostly present in the protein storage compartment of platelets. These granules contain some membrane-associated proteins (i.e., P-selectin) and several soluble proteins (i.e., fibrinogen and platelet-derived growth factor). Exocytosis of α-granules is considered a marker of platelet activation, which we evaluated by examining P-selectin expression through flow cytometry (Figure 2A).

MAPKs constitute a group of serine/threonine kinases that translate extracellular stimuli into cellular responses. MAPKs specific inhibitors or their knockout in mice evidenced the occurrence of ERK1/2, JNK1/2, and p38 MAPK in platelets and observed their participation in platelet activation [20]. All these kinases are activated by specific MAPK kinases. However, the pathophysiological roles of JNK1/2 and ERK1/2 in platelets are still unclear. A study demonstrated that the suppression of integrin α_IIb_β_3_ activation may be tangled with MAPK [21]. Moreover, ERK activation is regarded as an essential mediator in collagen-induced platelet aggregation [22]. Cytosolic phospholipase A_2_ catalyzes the release of AA to produce thromboxane A_2_, which is a crucial substrate of p38 MAPK activation induced by various platelet agonists [22]. These results discovered that the activation of either ERK1/2 and JNK1/2 or p38 MAPK can be significantly suppressed by Rut; this mechanism may be responsible for the high potency of Rut in antiplatelet effect in humans.

PI3K activation strongly contributes to platelet activation. All PI3K isoforms (i.e., α, β, γ, and δ) are expressed in platelets, and these enzymes are vital actors downstream of many platelet receptors including the collagen receptor glycoprotein (GP)VI or the ADP receptors P2Y12 and integrin α_IIb_β_3_ [23]. PI3Kβ was identified as a major isoform recruited for platelet signaling upon stimulation. The findings of the experiments involving the use of a selective inhibitor demonstrated the critical role of PI3Kβ in promoting arterial thrombosis, suggesting that this kinase can be a relevant target for antithrombotic therapy. In particular, PI3Kβ is particularly crucial downstream of the collagen receptor GPVI in terms of the control of PLCγ2 activation and Ca^2+^ mobilization [24]. Serine/threonine kinase Akt is a major and ubiquitous effector of PI3K. The three closely related isoforms of Akt (Akt1, Akt2, and Akt3) are expressed in human and mouse platelets [12]. Akt-knockout mice exhibited defects in platelet activation [25]. Hence, protein kinase-mediated Akt activation—particularly PI3Kβ—may be attractive targets for the antithrombotic drug development. In addition, both PI3K/Akt and MAPKs are mutually activated in platelets, and PKC is their upstream regulator [25]. Despite it being unknown whether or not the activators of Akt may participate on platelet activation, several candidates such as GSK3 (α and β isoforms) have been identified [25]; both isoforms are expressed in platelets, with GSK3β being the most abundant [26]. Inhibition of GSK3 appears to be necessary for full platelet responses following activation by various agonists. Platelet-specific PI3Kβ–deficient mice exhibited strong arterial thrombus instability under high shear stress due to the impairment of Akt activation and GSK3 inhibition within the growing thrombus [12]. However, the mechanism through which GSK3 regulates platelet activation remains unknown. Thus, the identification of the platelet substrates of GSK3 may provide promising candidates for the development of new antithrombotic drugs. Overall, the PI3K/Akt/GSK3β signal axis plays a vital role in platelet activation and thrombus growth and stability under high shear stress in vivo.

Increases in cyclic AMP and cyclic GMP in platelets activate their corresponding protein kinase A and G, which in turn regulate platelet activation via phosphorylating intracellular protein substrates, namely VASP (Ser157) and (Ser239), respectively [27]. Cyclic nucleotides elevation decreases the calcium flux and concurrently reduces the activity of cell membrane-bound calcium transporters that suppress the activation of PLC/PKC signaling. Chiou et al. [14] reported that Rut exerted a vasodilator effect on isolated mesenteric arteries through an endothelial NO-dependent mechanism. NO activates guanylate cyclase, thus increasing the intracellular cyclic GMP level. Nevertheless, neither SQ22536 nor ODQ substantially reversed Rut-mediated antiplatelet aggregation, and Rut exerted no significant effects on VASP^Ser157^ and VASP^Ser239^ phosphorylation, thus ruling out the possibility of the involvement of cyclic nucleotides/VASP signals in Rut-mediated antiplatelet activity. 

Hydroxyl radicals are the most reactive free radicals and can be formed from O_2_^−^ and H_2_O_2_ in the presence of metal ions such as copper and iron. In a biological system, hydroxyl radicals attack the cell membrane, causing membrane damage and destroying sugar groups and DNA base sequences, thus resulting in cell death and mutations [28]. Therefore, the scavenging activity of hydroxyl radicals is commonly examined to evaluate the ability of drugs to scavenge free radicals. In addition, hydroxyl radicals derived from platelet activation act as secondary stimulator on elevating the level of [Ca^2+^]i at the time of early phase of platelet activation [29], thus playing a vital role in regulating thrombus formation [29]. The ESR results of this current study directly evidence hydroxyl radicals’ scavenging properties of Rut in human platelets. Therefore, the Rut-mediated inhibition of thrombogenesis in vivo may involve, at least partly, the scavenging of free radicals formed in platelets.

Microvascular thrombosis is an ideal in animal model that is essential to understand the effectiveness of any test compounds for the treatment of this disease. In a microvascular thrombotic mouse model, using the fluorescein sodium, mesenteric venules were continuously irradiated in the entire experimental period, severely damaging the endothelium. Subsequently, subendothelial collagen is found to be powerfully triggered platelet adhesion and aggregation at the injury site, thus causing microvascular thrombosis [30]. Treatment with 0.7 and 1.5 mg/kg Rut prolonged the occlusion time in a dose-dependent manner; this data designates that platelet aggregation is a central factor in tempting microvascular thrombosis. Therefore, Rut may be considered to be a potent alkaloid compound for treating thromboembolic disorders.

## 4. Materials and Methods

### 4.1. Chemicals and Reagents

Rut (>98%); *N*-[2-[[3-(4-bromophenyl)-2-propen-1-yl]amino]ethyl]5-isoquinolinesulfonamide dihydrochloride (H-89); 2,3,9,10,11,12-hexahydro-10R-methoxy-2,9-dimethyl-1-oxo-9S, 12R-epoxy-1H-diindolo[1,2,3-fg:3′,2′,1′-kl]pyrrolo[3,4-i][1,6]benzodiazocine-10-carboxylic acid; and methyl ester (KT5823) were purchased from Cayman Chem (Ann Arbor, MI, USA). Collagen (type I), luciferin–luciferase, AA, 9,11-dideoxy-11α,9α-epoxymethanoprostaglandin (U46619), 9-(tetrahydro-2-furanyl)-9H-purin-6-amine (SQ22536), ODQ, heparin, PGE_1_, NTG, phenylmethylsulfonyl fluoride, sodium orthovanadate, sodium pyrophosphate, aprotinin, leupeptin, sodium fluoride, PDBu, and thrombin were purchased from Sigma (St. Louis, MO, USA). The anti-phospho-p38 MAPK (Thr^180^/Tyr^182^) polyclonal antibody (pAb) was purchased from Affinity (Cincinnati, OH, USA). Anti-phospho-JNK (Thr^183^/Tyr^185^), anti-PLCγ2, anti-phospho-p44/p42 ERK (Thr^202^/Tyr^204^), anti-phospho-(Ser) PKC, and anti-phospho-PI3 kinase p85 (Tyr^458^)/p55 (Tyr^199^) substrate pAbs and anti-Akt, anti-p38 MAPK, anti-PLCγ2, and anti-PI3K p85 (19H8) monoclonal antibodies (mAbs) were purchased from Cell Signaling (Beverly, MA, USA). Anti-phospho-GSK3α/β and anti-GSK3α/β mAbs were purchased from Santa Cruz Biotechnology (Santa Cruz, CA, USA). The protein assay dye reagent concentrate was purchased from Bio-Rad Laboratories (Hercules, CA, USA), and anti-phospho-Akt (Ser^473^) pAb was purchased from BioVision (Mountain View, CA, USA). Anti-VASP (phospho Ser^157^), anti-VASP (phospho Ser^239^), and anti-VASP pAbs were purchased from GeneTex (Irvine, CA, USA). Fura 2-AM was obtained from Molecular Probes (Eugene, OR, USA). The FITC-anti-human CD42P (P-selectin) mAb was obtained from BioLegend (San Diego, CA, USA). 5,5-Dimethyl-1 pyrroline N-oxide (DMPO) was purchased from Enzo Life Sciences (Farmingdale, NY, USA). Hybond-P polyvinylidene difluoride membranes, enhanced chemiluminescence Western blotting detection reagent, horseradish peroxidase–conjugated donkey anti-rabbit immunoglobulin G (IgG), and sheep anti-mouse IgG were obtained from Amersham (Buckinghamshire, UK). Rut was dissolved in 0.1% DMSO and stored at 4 °C until used.

### 4.2. Platelet Aggregation, ATP-Release Reaction, and Cytotoxicity Assay

This study conformed to the directives of the Declaration of Helsinki and was approved by the Institutional Review Board of Taipei Medical University (TMU-JIRB-N201812024). All human volunteers who participated in this study were signed in the consent forms. Washed human platelets (3.6 × 10^8^ cells/mL) were prepared as described previously [31]. Blood was mixed with acid/citrate/glucose (9:1, v/v). After centrifugation, the supernatant (PRP) was supplemented with EDTA (2 mM) and heparin (6.4 U/mL) for 5 min and centrifuged at 500× *g* for 10 min. The platelet pellet was suspended in 5 mL Tyrode’s solution for 10 min at 37 °C. After centrifugation of the suspension at 500× *g* for 10 min, the washing procedure was repeated. The washed platelets were finally suspended in Tyrode’s solution containing bovine serum albumin (3.5 mg/mL). The platelet count was monitored using a Coulter counter (Beckman Coulter, Miami, FL, USA). The final concentration of Ca^2+^ in Tyrode’s solution was 1 mM. Solvent control (0.1% DMSO) or various concentrations of Rut (1–100 µM) were preincubated with platelets for 3 min before stimulation with various agonists, namely collagen, thrombin, U46619, and AA. ATP release was examined using a Hitachi Spectrometer F-7000 (Tokyo, Japan) in accordance with the manufacturer’s instructions. In addition, washed platelets were preincubated with Rut (2.5–100 µM) or 0.1% DMSO for 20 min at 37 °C. An aliquot of the supernatant (10 µL) was deposited on a Fuji Dri-Chem slide (LDH-PIII; Fuji, Tokyo, Japan), and absorbance was read at a wavelength of 540 nm by using an ultraviolet-visible spectrophotometer (UV-160; Shimadzu, Kyoto, Japan). The highest level of LDH was noted in positive triton-treated platelets.

### 4.3. Surface P-Selectin Expression and Intracellular [Ca^2+^]i Mobilization

Washed platelets (3.6 × 10^8^ cells/mL) were preincubated with either solvent control (0.1% DMSO) or Rut (2.5 and 5 µM) and FITC-conjugated anti-P-selectin mAb (2 µg/mL) for 3 min, followed by stimulation with collagen (1 µg/mL). Subsequently, the suspensions were used to assay for fluorescein-labeled platelets by using a flow cytometer (FAC Scan system; Becton Dickinson, San Jose, CA, USA). Data were collected from 50,000 platelets per in each experimental group, and the platelets were identified based on their characteristic forward and orthogonal light-scattering profiles. Independent experiments (*n* = 4) were performed to ensure reproducibility of all the all experiments. Citrated whole blood was subject to centrifugation at 120× *g* for 10 min to measure [Ca^2+^]i mobilization, the supernatant was incubated with Fura 2-AM (5 µM) for 1 h. Platelet suspensions (3.6 × 10^8^ cells/mL) were prepared as described in the previous section, which were adjusted to 1 mM Ca^2+^. The relative intracellular Ca^2+^ concentration was measured using a Hitachi Spectrometer F-7000 (Tokyo, Japan) at the excitation wavelengths of 340 and 380 nm and the emission wavelength of 500 nm [32].

### 4.4. Immunoblotting

Washed platelets (1.2 × 10^9^ cells/mL) were preincubated with solvent control (0.1% DMSO), Rut (2.5 and 5 µM), PGE_1_ (20 nM), or NTG (20 µM) and then stimulated with or without collagen (1 µg/mL) for 5 min. After the collagen stimulation, the platelets were directly resuspended in 200 µL lysis buffer and subjected to. centrifugation at 5000× *g* for 5 min, and supernatants were collected. An 80 µg measure of protein from the supernatants was separated through 12% sodium dodecyl sulfate-polyacrylamide gel electrophoresis. Protein concentrations were determined using the Bradford protein assay (Bio-Rad, Hercules, CA, USA). The targeted proteins were deducted by using their respective primary antibodies. The optical density of protein bands was quantified using a video densitometer and Bio-profil Biolight software, version V2000.01 (Vilber Lourmat, Marne-la-Vallée, France). The relative protein expression was calculated after normalization to the total protein of interest.

### 4.5. Measurement of Hydroxyl Radicals through ESR Spectrometry

ESR spectrometry was used to measure hydroxyl radicals by using a Bruker EMX ESR spectrometer, as described previously [33]. Briefly, platelet suspensions (3.6 × 10^8^ cells/mL) were preincubated with solvent control (0.1% DMSO) or Rut (2.5 and 5 µM) for 3 min before adding collagen (1 µg/mL). The reaction was allowed to proceed for 5 min before adding DMPO (100 µM). The ESR spectrometer was operated at a power of 20 mW and 9.78 GHz, and a scan range of 100 G and a receiver gain of 5 × 10^4^ were applied. The ESR signal amplitude was quantified using the WIN-EPR, version 921201, supplied by BRUKER-FRANZEN Analytik GmbH (Bremen, Germany). 

### 4.6. Measurement of Microvascular Thrombosis in Mice

The method used to establish a thrombogenic animal model in this experiment conformed to the Guide for the Care and Use of Laboratory Animals (8th edition, 2011), and we received an affidavit of approval for the animal use protocol from Taipei Medical University (LAC-2019-0365). Male ICR mice (6 weeks) were anesthetized using a mixture containing 75% air and 3% isoflurane maintained in 25% oxygen; their external jugular veins were then cannulated using a PE-10 tube for administering the dye and drugs intravenously [33]. Venules (30–40 µm) were irradiated at a wavelength of <520 nm to produce a microthrombus. Two Rut doses (0.7 and 1.5 mg/kg) were intraperitoneally administered 1 min following the administration of sodium fluorescein (15 µg/kg), and the time required for the thrombus to occlude the microvessel (OT) was recorded.

### 4.7. Measurement of Tail Bleeding Time in Mice

Bleeding time was measured in male ICR mice through a tail-vein transection model. To this, mice were sedated and intraperitoneally administered Rut (0.7 and 1.5 mg/kg) or 0.1% DMSO for 30 min. Following injury, tails were directly placed into normal saline at 37 °C and the bleeding time was recorded until the bleeding was totally stopped.

### 4.8. Statistical Analysis

The data are presented as the mean ± standard error of the mean. Values of *n* refer to the number of experiments that were conducted in different blood donors. Significant differences among the experimental groups in mice were analyzed using one-way analysis of variance (ANOVA) with the Student–Newman–Keuls method as the posthoc test to control for family-wise Type-I error. Variations in the experimental setup were calculated using one-way ANOVA. Significant differences between the groups were compared using the Student–Newman–Keuls method; *p* < 0.05 indicated statistical significance. Statistical analyses were performed using SAS Version 9.2 (SAS Inc., Cary, NC, USA).

## 5. Conclusions

Balanced diet and healthy lifestyle are key variable risk factors for the primordial prevention of CVDs. The results of this study offer new understandings into the role of Rut in human platelet activation. In particular, Rut effectively inhibited platelet activation by hindering the PI3K/Akt/GSK3β and MAPK pathways through a cyclic nucleotides/VASP-independent mechanism. Rut inhibits the P-selectin expression, ATP release, and [Ca^2+^]i, which in turn eventually inhibit platelet aggregation. The results of the study may exhibit the potential of Rut in therapeutic application for thrombotic diseases.

## Data Availability

All data generated or analyzed during this study are included in this published article.
